# Improved detection of house infestations with triatomines using sticky traps: a paired-comparison trial in the Argentine Chaco

**DOI:** 10.1186/s13071-020-3891-z

**Published:** 2020-01-14

**Authors:** Gustavo Fabián Enriquez, María Carla Cecere, Julián Antonio Alvarado-Otegui, Alejandra Alvedro, María Sol Gaspe, Mariano Alberto Laiño, Ricardo Esteban Gürtler, Marta Victoria Cardinal

**Affiliations:** 1 Laboratorio de Eco-Epidemiología, Departamento de Ecología, Genética y Evolución, Facultad de Ciencias Exactas y Naturales, Universidad de Buenos Aires, Buenos Aires, Argentina; 2 Instituto de Ecología, Genética y Evolución (IEGEBA), Universidad de Buenos Aires, CONICET, Buenos Aires, Argentina

**Keywords:** Chagas disease, *Triatoma infestans*, Vector control, Detection methods, Surveillance

## Abstract

**Background:**

We conducted a matched-pairs trial of three methods for detecting house infestation with triatominae bugs in a well-defined endemic rural area in the Argentine Chaco.

**Methods:**

The three methods included a simple double-sided adhesive tape (ST) installed near host resting sites; timed-manual collections with a dislodging aerosol (TMC, the reference method used by vector control programmes), and householders’ bug notifications (HN). Triatomine infestations were evaluated in 103 sites of 54 houses, including domiciles, kitchens and storerooms.

**Results:**

In domiciles where *Triatoma infestans* was collected, sensitivity of each single method decreased from 79% by ST and 77% by HN, to 57% by TMC, and increased to 92% when ST was combined with HN. In peridomestic kitchens and storerooms, TMC was relatively as sensitive as ST and significantly more sensitive than HN. On average, the number of bugs recovered by ST was 0.94 times that collected by TMC. The ST mainly collected early-instar nymphs whereas TMC yielded late (larger) stages. Triatomines caught by ST had significantly lower mean weight-to-length ratios and lower blood-feeding rates than those caught by TMC, suggesting the ST intercepted and trapped vectors seeking a blood meal host.

**Conclusions:**

The ST may effectively replace TMC for detecting *T. infestans* in domiciles, and is especially apt for early detection of low-density domestic infestations in the frame of community-based surveillance or elimination programmes; decision making on whether an area should be targeted for full-coverage insecticide spraying, and to corroborate that extant conditions are compatible with the interruption of vector-borne transmission.
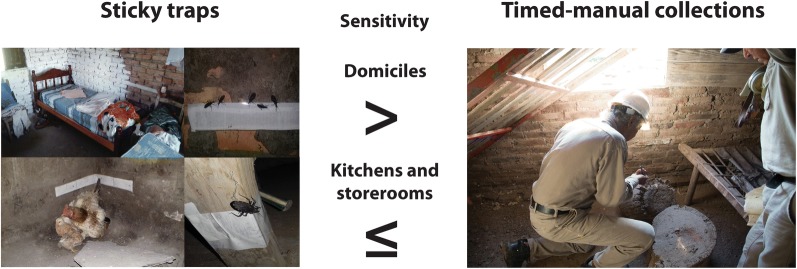

## Background

Chagas disease continues to be one of the main neglected tropical diseases in Latin America [1]. In the absence of a vaccine against the etiological agent of the disease, *Trypanosoma cruzi*, prevention of vector-borne transmission mainly relies on the detection of house infestations with triatomine bugs followed by residual insecticide spraying of domestic and peridomestic habitats [2–5]. The success of triatomine control efforts is threatened by the frequent occurrence of persistent peridomestic foci [6–9]; the emergence of resistance to pyrethroid insecticides [10], and/or house invasion of triatomines from sylvatic sources [11–14]. Sustained vector surveillance and control are required for the effective interruption of the domestic transmission of *T. cruzi* in highly endemic rural areas [15].

Assessments of house infestation with triatomine bugs have classically been conducted using timed-manual collections (TMC) in the hands of skilled personnel assisted or not with a dislodging spray [16, 17]. This method has limited sensitivity [16, 18–21], especially at the low triatomine densities that typically prevail after community-wide insecticide spraying campaigns. The limited sensitivity of TMC affects the reliability of house infestation indices and vector control decisions.

Given the substantial operational costs associated with TMC [22] and the absence of a “gold standard method” for establishing house infestation status, several passive devices have been developed and trialed under field conditions [20–23]. Most of these devices offer a dry refuge for triatomines; some are resistant to outdoor conditions [24], and may include attractants or chemical baits and serve as traps [25, 26]. Community-based vector surveillance conducted by householders, either in the form of bug notifications (HN) or bug collections (supplemented or not with a passive device), frequently performed similar to or better than TMC in domestic habitats [21, 27–31]. The validity of householder-based triatomine surveillance is affected by the vagaries of voluntary participation and shifting motivations, the skill of local residents, triatomine density, and whether householders provide proof of house infestation by returning triatomines to the designated receptors or not [18, 27, 30].

Double-sided sticky tape (sticky traps, ST) adhered to specific substrates or habitats used by the target organisms has been used for arthropod detection in agricultural pest management programmes [32], and for monitoring of mosquito-borne human disease [33, 34]. The ST combined with a live bait held in a plastic bottle (i.e. the Noireau trap) revealed the occurrence of multiple triatomine species in sylvatic habitats that are difficult to inspect by TMC, such as palm tree crowns or burrows [35–38]. Sticky traps have been used in Bolivia for surveillance of domestic infestations with *T. infestans*, the main vector of Chagas disease in the Southern Cone countries [39], and in the Argentine Chaco to evaluate the dispersal of several triatomine species [40]. A cardboard box containing both a glue and a semiochemical bait has been tested for assessment of domestic and peridomstic bug infestations [25]. Despite of the frequent use of sticky tapes or glues in various formats and promising scope, a rigorous assessment of the relative performance of ST and TMC for detecting house infestations with triatomines is still lacking.

As part of a broader programme including renewed control interventions to suppress vector-borne transmission, in this study we evaluated the ability of ST, TMC and HN for detecting bug infestations in domiciles, kitchens and storerooms of a well-defined endemic rural area in the Argentine Chaco. We also assessed whether both methods yielded similar estimates of bug abundance, stage distribution, nutritional status, and blood-feeding frequency. Our results are relevant for both vector surveillance and elimination programmes, and for decision making on whether the affected communities should be targeted for full-coverage insecticide spraying.

## Methods

### Study area

Field work was conducted in three adjacent rural villages (La Unión, La Esperanza and Campo Florido) located in the municipality of Juan José Castelli (25° 57′00′′ S, 60° 37′00′′ W), Chaco Province, Argentina. The study area has hot, humid summers concentrating most of the scarce rainfall, and mild, dry winters. The three villages comprised 99 dwellings inhabited by creoles. Most houses had mud walls, metal or thatched roofs, and included peridomestic structures that housed domestic animals. A house compound encompassed the domicile and all sites within the peridomestic area.

Local health officials and primary healthcare agents reported the three study villages had ongoing infestations as of late 2018. All the study houses had been last sprayed with pyrethroid insecticides by the vector control programme in approximately 2014. High levels of pyrethroid resistance in *T. infestans* populations had been recorded in nearby rural villages [41].

### Study design

We conducted a matched-pairs trial of triatomine detection methods in two successive phases. In the first phase, we performed a cross-sectional survey of house infestation by using TMC and HN in the three study villages in 2018. We excluded from the paired analysis 19 houses closed during the survey; one TMC-positive house that was repeatedly vacant after the initial evaluation, and four households that did not allow the installation of ST in domiciles. In the second phase, we tested the performance of ST in all the 39 TMC-positive houses detected and in a random sample of 15 (out of 40) TMC-negative houses over December 2018.

### Vector methods

For TMC, two skilled personnel from the Chaco Vector Control Programme searched for triatomines in all domestic and peridomestic sites of 80 occupied houses (19 were closed during the survey) using a dislodging aerosol (0.2% tetramethrin; Espacial, Buenos Aires, Argentina) in October–November 2018. The unit of search effort at each site was one person during 15 min. Bug abundance per site was calculated as the number of bugs collected by TMC per unit of search effort.

During TMC searches, a member of the research group interviewed a household resident on the current presence of triatomines in domiciles and in other frequently infested peridomestic outhouses (kitchens and storerooms, see below) and the usual resting places of domestic animals at each house. We also asked whether triatomines were seen during the previous year, and dry specimens of different species were shown in order to help the identification of the reported species. The former information was used as a house and site infestation index denominated householders’ bug notification (HN) [30]. The terms “infested” or “positive” were taken to mean the finding of any live adult or nymph of *T. infestans* in specified sites (for TMC and ST) or a householder’s positive report of the finding or sighting of triatomines in specified sites (for HN). Householders provided no information on triatomine presence in five houses (including 9 sites) in which we subsequently deployed sticky traps.

**Fig. 1:**
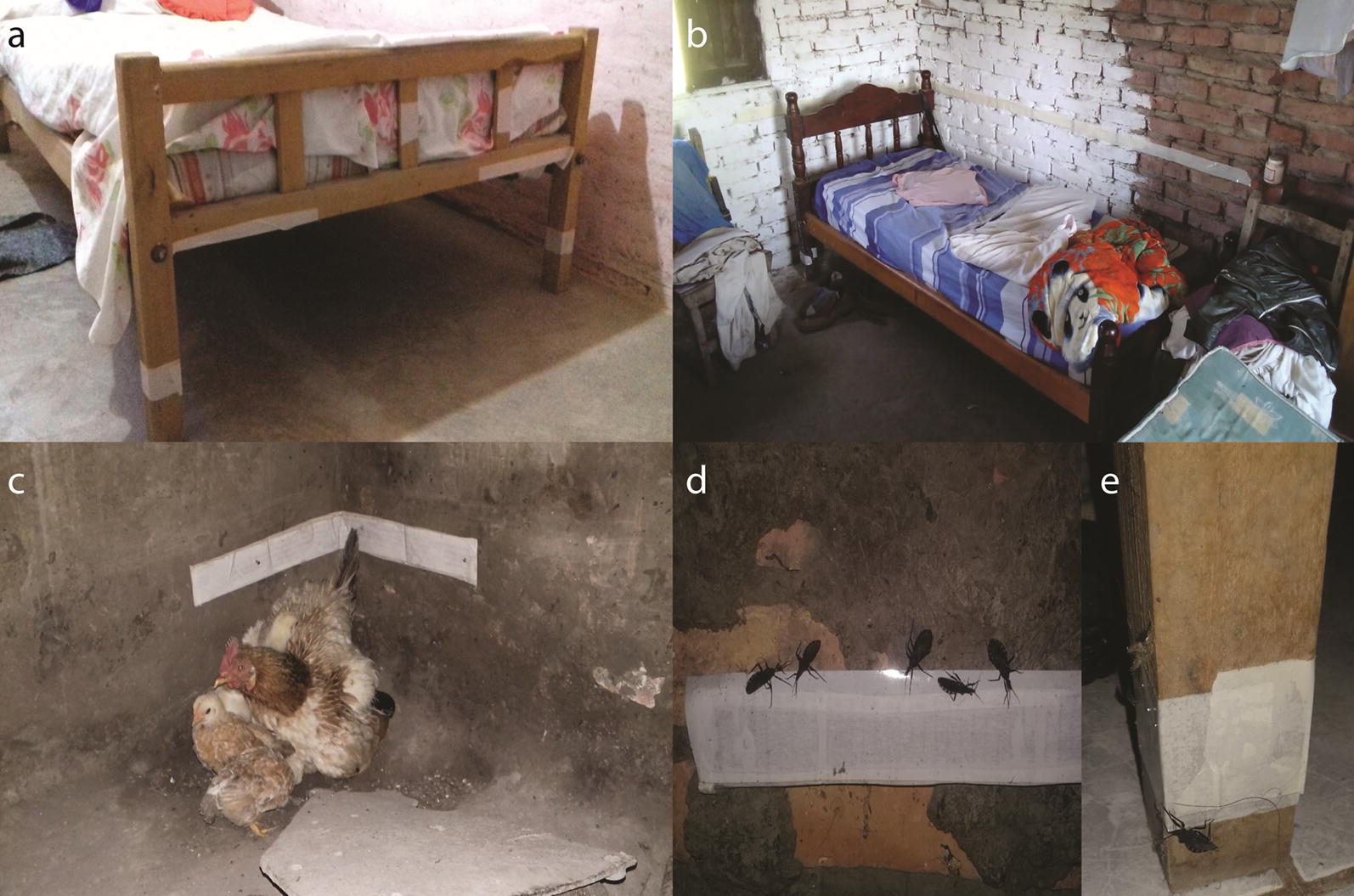
Sticky traps installed in domiciles (**a**, **b**) and kitchens (**c**). Adults of *Triatoma infestans* captured by sticky traps (ST) installed on a wall (**d**) and a bed leg (**e**) in domiciles

The ST consisted of a 5 cm-wide, white, double-sided adhesive tape (Plasto^®^, São Paulo, São Paulo, Brazil), which has been successfully used for trapping several triatomine species including *T. infestans* [11, 36]. It was fixed to regular surfaces (cement, wood or mud) or nailed to a cardboard if the surface was irregular, close to the resting sites of domestic hosts or where triatomine feces were observed (Fig. 1). The STs were only deployed in ecotopes with at least three walls and a roof (i.e. domiciles, kitchens and storerooms) in order to prevent the early loss of tape adherence due to moist or dust. Therefore, chicken coops or nests, pig corrals and goat corrals were excluded from the current trial, and our reported estimates of house infestation rates only encompass a fraction of the house compound. In domiciles, the ST covered the entire perimeter of each leg of all beds in use and the adjacent walls (Fig. 1). The average time spent installing each ST was 37 person-min per house (standard deviation, SD = 15) or 23 person-min per site (SD = 11). The average length of adhesive tape installed per site was much greater in domiciles (mean ± SD, 6.7 ± 3.4 m) than in storerooms (1.2 ± 0.9 m) and kitchens (0.7 ± 0.6 m). In total, we used 388 m of adhesive tape. The STs were set up immediately after or within 2 days of conducting TMC searches in 20 houses, and approximately 23 days after TMC in 34 houses in December 2018.

Each ST was inspected for triatomines 1, 3 and 5 days post-installation in 42 houses, and 3–4 days post-installation in 12 houses that could not be accessed after a heavy rainfall. The evaluation of ST was restricted to 5 days post-installation because preliminary observations indicated that it lost adhesiveness thereafter. For ST, bug abundance per site was calculated as the total number of bugs collected across the exposure period. In total, both ST and TMC provided a diagnosis of triatomine infestation in 103 sites (mean ± SD of the number of sites per house, 1.9 ± 0.68) from 54 houses.

### Blood-feeding frequency and nutritional status

To depress their metabolism until examination in the field laboratory, the collected triatomines were kept at 10–12 °C in plastic bags labeled with unique codes for house and collection site. The insects were identified taxonomically and counted according to species, stage, and sex as described elsewhere [8]. Each triatomine was individually weighed in an electronic balance (precision of 0.1 mg; OHAUS, Pine Brooks, NJ, USA) and measured from clypeus to abdominal tip with a hand-held caliper (accurate to 0.02 mm) as described in Ceballos et al. [42]. The log-transformed weight-to-length (W/L) ratios of 109 bugs captured by TMC and in 37 by ST were used as indices of nutritional status [43].

All fourth- or fifth-instar nymphs and adults were individually examined by abdominal compression for the presence of colorless urine, used as an index of recent blood-feeding (nearly 24 h before bug capture) [44]. The proportion of *T. infestans* that fed during the preceding night was estimated as the observed proportion of fourth- and fifth-instar nymphs with colorless urine multiplied by a temperature-dependent correction factor (*c *= 0.0533, t= 0.585) and the (uncorrected) proportion of adult bugs with colorless urine [43, 44]. The mean temperature (t, in °C) from 20:00 to 6:00 h was used as the daily correction factor *c* for triatomines captured shortly after. Ambient temperatures were recorded using data loggers (HOBO® H08; Onset Computer Corporation, Bourne, MA, USA). The mean temperature throughout the study was 26.6 °C (SD = 6.5 °C; range, 12.2–42.5 °C). The frequency of colorless urine was assessed in 77 (68%) *T. infestans* captured by TMC (11 from domiciles and 66 from peridomiciles) and in 34 (32%) by ST (12 from domiciles and 22 from peridomiciles); it was not determined in 43 bugs that had scarce contents. The feeding interval (in days) was calculated as the inverse of the proportion of vectors that fed during the preceding night.

### Data analysis

The sensitivity of each detection method was calculated as the proportion of truly infested houses (or sites) that tested positive using a given method. Given the absence of a gold standard for house or site infestation, this calculation used in the denominator two alternative definitions of a truly infested house (or site). In definition 1, the most conservative, the denominator only included inspected houses (or sites) classified as infested because of the finding of at least one live triatomine by ST or TMC. In definition 2, the denominator included the total number of inspected houses (or sites) that were positive by at least one of the three methods used (ST, TMC and HN); this was a less stringent approximation to true local infestation status since householders were not required to provide any direct evidence of infestation (i.e. live bugs, exuviae or eggs). Houses (or sites) with missing data for HN were excluded from the relevant calculations, except for overall infestation rates as determined by all methods combined. We assumed that observations within the same house compound were independent and tested for the significance of the observed differences between methods using exact McNemar’s Chi-square test. In addition to paired comparisons between individual methods, we tested the combined result of ST and householders’ bug notification (ST-HN). On a posteriori basis we calculated the power of the tests used in paired comparisons.

The relative sampling efficiency of ST to TMC was measured by the log-transformed ratios of the number of bugs collected in each site by ST (*x*) and by TMC (*y*) {log_10_[(*x* + 1)/(*y* + 1)]} [45, 46]. We tested whether the relative sampling efficiency varied with the total number of bugs collected by both methods, taken as an approximation of the actual bug population abundance. We used Fisher’s exact tests to examine differences in the stage structure of the bug populations collected by ST and TMC, and the Wilcoxon test to compare the log-transformed W/L ratios of bugs collected by each method. All analyses were conducted as implemented in Stata 15.1 [47].

## Results

The prevalence of infestation at house level varied from 35 to 50% depending on the detection method considered (Table 1). All methods combined detected infestations in 57% of the inspected houses. Bug colonies (i.e. with live nymphs) were detected by TMC or ST in 10 (71%) domiciles and 16 (94%) kitchens or storerooms. Using definition 1, the sensitivity using a single method decreased from 78% as determined by ST and 70% by TMC, to 54% by HN, and increased to 88% when ST was combined with HN (Table 1). All pairwise comparisons at house level were statistically non-significant except between ST-HN and TMC, which was marginally significant (*χ*^2^ = 4.00, *df* = 1, *P* = 0.077) (Additional file 1: Table S1). The power of the latter test reached 0.51, whereas for other comparisons it was very low (< 10%). The same ranking of methods held when the denominator of sensitivity included houses positive by any of the three methods employed (definition 2, last column in Table 1).

**Table 1:** Infestation with *Triatoma infestans* and sensitivity of sticky traps (ST), householdersʼ bug notifications (HN), timed-manual collections (TMC) and ST combined with HN

Level	Detection method	Percentage of infestation (No. positive/No. evaluated)	Sensitivity (%) according to houses or sites positive by	
			ST or TMC (No. positive)^c^	ST, TMC or HN (No. positive)^d^
House^a^				
	ST	39 (21/54)	78 (21/27)	68 (19/28)
	HN^b^	35 (17/49)	54 (13/24)	61 (17/28)
	TMC	35 (19/54)	70 (19/27)	61 (17/28)
	ST-HN	50 (27/54)	88 (21/24)	89 (25/28)
	All methods combined	57 (31/54)	100 (27/27)	100 (28/28)
Domiciles				
	ST	22 (11/51)	79 (11/14)	61 (11/18)
	HN^b^	32 (15/47)	77 (10/13)	83 (15/18)
	TMC	16 (8/51)	57 (8/14)	39 (7/18)
	ST-HN	33 (17/51)	92 (12/13)	94 (17/18)
	All methods combined	37 (19/51)	100 (14/14)	100 (18/18)
Kitchens and storerooms				
	ST	21 (11/52)	65 (11/17)	64 (9/14)
	HN^b^	6 (3/47)	21 (3/14)	21 (3/14)
	TMC	27 (14/52)	82 (14/17)	86 (12/14)
	ST-HN	21 (11/52)	64 (9/14)	64 (9/14)
	All methods combined	33 (17/52)	100 (17/17)	100 (14/14)

^a^House level only encompasses domiciles, storerooms and kitchens

^b^Householders provided no information on triatomine presence in five houses (including four domiciles and five kitchens and storerooms)

^c^Using Definition 1, the denominator only included houses (or sites) classified as infested because of the finding of at least one live triatomine as determined by ST or TMC

^d^Using Definition 2, the denominator included the total number of houses (or sites) that were positive as determined by at least one of the three methods used (ST, TMC and HN)

At site level, the percentage of infested sites increased from 16 to 33% in domiciles, and from 6 to 27% in kitchens and storerooms (Table 1). In domiciles, the sensitivity of a single detection method (under definition 1) decreased from 79% as determined by ST and 77% by HN, to 57% by TMC, and increased to 92% when ST was combined with HN. HN and ST-HN were significantly more sensitive than TMC for detecting domestic infestations (*χ*^2^ = 6.40, *df* = 1, *P* = 0.022; *χ*^2^ = 6.23, *df* = 1, *P* = 0.023, respectively) (Additional file 1: Table S1). Only for domiciles was the ranking of detection methods slightly affected by the definition of a truly infested house.

In kitchens and storerooms, sensitivity decreased from 82% as determined by TMC, 64–65% by ST or ST-HN, to 21% by HN (definition 1, Table 1). TMC was as sensitive as ST and significantly more sensitive than HN (*χ*^2^ = 9.00, *df* = 1, *P* = 0.004) (Additional file 1: Table S1). There were no significant differences between other pairwise comparisons, except for HN *versus* ST in kitchens and storerooms (*χ*^2^ = 6.00, *df* = 1, *P* = 0.031) (Additional file 1: Table S1, Additional file 2: Table S2). The only comparisons in which the power of the tests exceeded 70% were those that were statistically significant; in the remainder the power was very low.

The study ecotopes had low median abundances of *T. infestans*. In domiciles, both methods captured 2 triatomines per site, whereas in peridomestic sites TMC caught 3.5 times as many bugs as ST (median: 7, Q1–Q3: 1–15 *vs* median 2, Q1–Q3: 1–6). Ten of the 14 HN-negative sites where triatomines were caught at least by TMC had low bug densities (median: 4 triatomines per site, Q1–Q3: 1–7). The other four HN-negative sites were TMC-negative and ST-positive (median: 1, Q1–Q3: 1–1).

The ST detected *T. infestans* in 55% (17/31) of all sites positive by either ST or TMC one day post-installation. The cumulative frequency of positive sites rose to 68% (21 out of 31) and 71% (22 out of 32) at 3 and 5 days post-installation, respectively. Only one site was initially found ST-positive at 5 days post-installation; triatomine feces and a live small lizard (*Mabuya frenata*, known to prey on insects) had been registered on the ST.

**Fig. 2:**
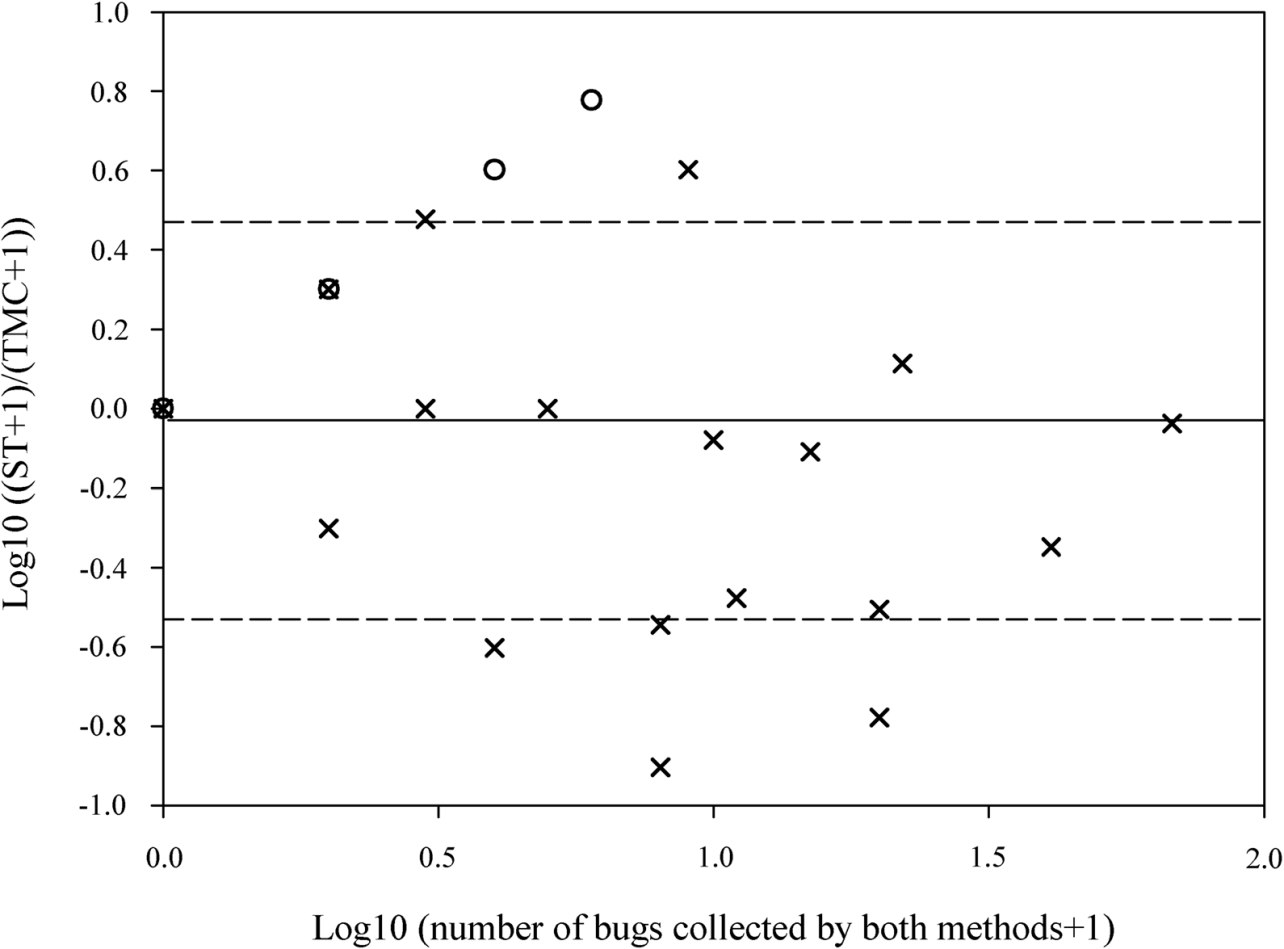
Log-ratio between the number of *Triatoma infestans* captured by sticky traps (ST) and timed-manual collections (TMC) in relation to the total number of *Triatoma infestans* caught by both methods per site. *Key*: solid line, the mean log-ratio; dashed lines, the expected range for 95% of individuals log-ratios; circles, sites negative per TMC;  × , sites positive per TMC

The log-transformed ratios of the numbers of bugs captured by ST and TMC correlated negatively and highly significantly with the total number of bugs captured (Fig. 2; *r* = − 0.306, *P *< 0.001). The antilog of the mean log ratio was − 0.03 (SE = 0.03), and the geometric mean ratio was 0.94 (95% CI 0.81–1.01). Hence, on average, the number of bugs recovered by ST was 0.94 times the number collected previously by TMC.

**Fig. 3:**
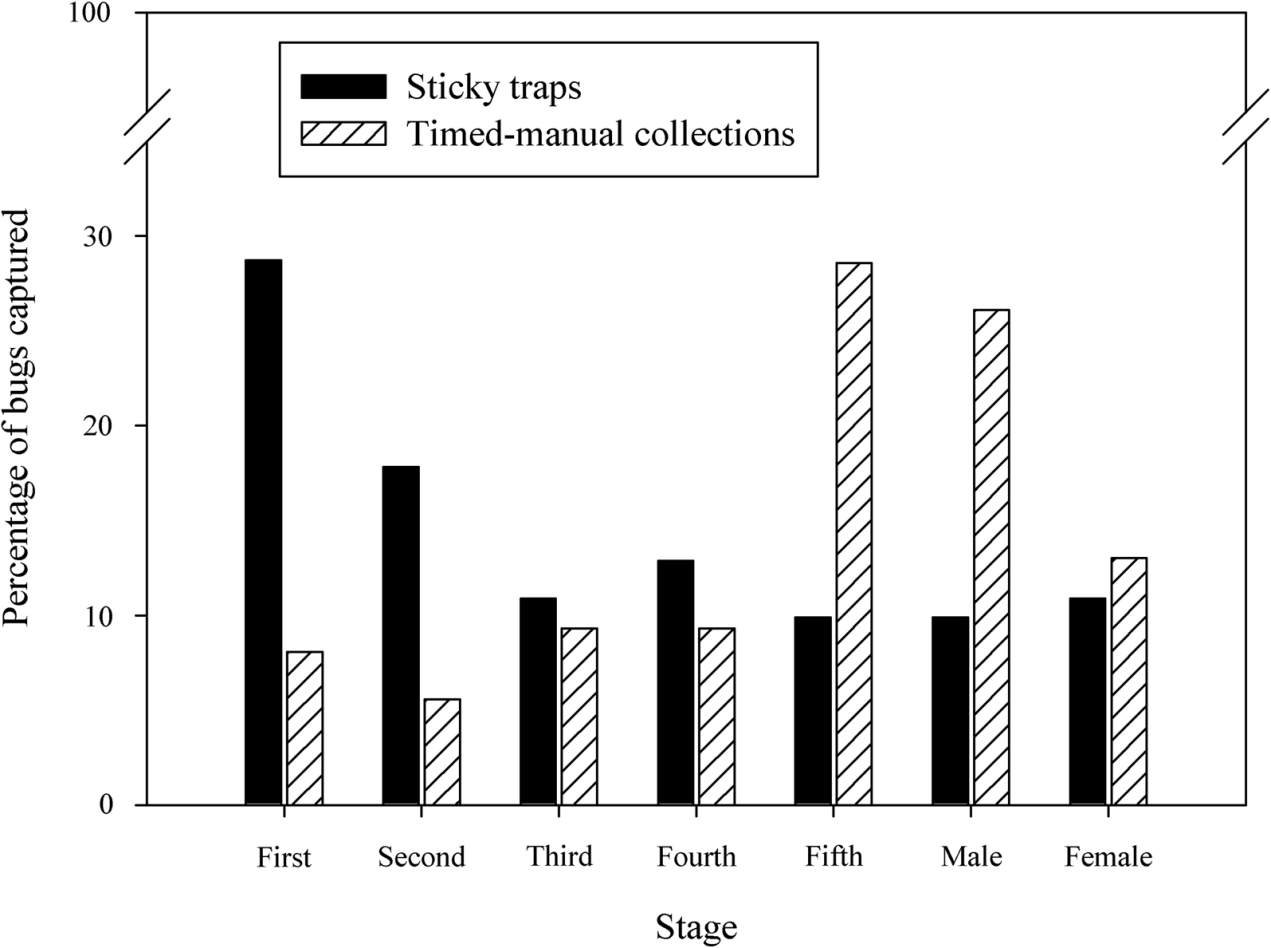
Stage structure of *Triatoma infestans* according to sticky traps (ST) and timed-manual collections (TMC)

The stage structure of *T. infestans* collected by TMC (*n* = 161) differed significantly from the one revealed by ST (*n* = 102) (Fig. 3; Fisher’s exact test, *df* = 3; *P *< 0.01). The ST captured more first- or second-instar nymphs (50%) than other stages (range, 10–13%), whereas TMC captured more adults and fifth-instars (68%) than other stages (< 10% for each of the other instars). The ST also caught mosquitoes, ticks and non-hematophagous arthropods (mainly flies, spiders, and cicadas) but no other triatomine species. Timed-manual collections collected 41 *Triatoma sordida* in 10 (19%) houses, mostly in ecotopes used by chickens (which were excluded from the comparison trial), and in a storeroom (a colony of six insects).

The temperature-adjusted proportion of bugs with colorless urine captured by ST was nearly half (5%, 95% CI 2–20%) of that recorded in triatomines captured by TMC (9%, 95% CI 5–18%). Only one triatomine captured in domiciles (by TMC) had colorless urine (i.e. recently blood-fed). The blood-feeding intervals of bugs collected by ST and TMC were 20 and 11 days, respectively.

The log-transformed W/L ratios significantly differed between capture methods in females and fifth-instar nymphs (Wilcoxon test, *P* = 0.037 and *P* = 0.007, respectively), but no significant differences were detected in males and fourth-instars (Wilcoxon test, *P* = 0.439 and *P* = 0.160, respectively; Table 2).

**Table 2:** Log-transformed weight-to-length ratios (log W/L) of *Triatoma infestans* captured by sticky traps (ST) and timed-manual collections (TMC)

Detection method	Median log (W/L) of bugs (SD; *n*)			
	Fourth-instar	Fifth-instar	Female	Male
ST	1.27 (0.77; 10)	1.65 (0.26; 7)**	2.40 (0.23; 10)*	2.08 (0.27; 10)
TMC	1.94 (0.43; 14)	2.62 (0.62; 40)**	2.57 (0.28; 17)*	2.17 (0.24; 38)

*Abbreviation*: n, number of bugs examined

*P = 0.037, **P = 0.007

## Discussion

Our study documents that the ST was more sensitive than TMC to detect *T. infestans* in domiciles (79 *vs* 57%, by definition 1) in a context where low-density infestations prevailed, and was less sensitive than TMC in kitchens and storerooms (65 *vs* 82%). In general, HN performed much better in domiciles than in peridomestic outhouses (77 *vs* 21%), and householders missed some light domestic infestations revealed by ST or TMC, as expected [28]. The ranking of detection methods was quite robust to the exact definition of a truly infested house. The combination of ST and HN returned the maximum values of infestation in domiciles but not in peridomestic outhouses, where each of these methods performed worse than TMC. Although the use of ST is not new to the world of Chagas disease vectors, here we offer the first estimates of their relative capacity to detect domestic and peridomestic infestations with a major vector species.

The relative sampling efficiency of ST did not differ from TMC and weakly declined with total bug abundance at site level. Because TMC searches preceded the exposure of ST, the former may have removed a large fraction of low-density bug populations and possibly lead to discordant results between methods. Similarly, the 3- to 20-day lag between TMC searches and ST exposure may have contributed to discordant results if triatomines invaded the premises during the intervening period. Perhaps this was the case in six TMC-negative, ST-positive domiciles where mostly adult *T. infestans* were captured, or they were simply low-density infestations undetectable by TMC. In two of these houses, however, the residents had reported triatomines at the time of TMC, suggesting these were undetected local infestations missed by TMC. Although the dust and high temperatures prevalent in kitchens and storerooms may cause loss of adhesiveness, all STs installed there were tested in each inspection and they remained sticky until the end of the observation period. An alternative explanation could be the presence of predators, especially chickens, which could prey on the glued triatomines.

Householders’ bug notifications (HN) were significantly more sensitive than TMC and were similar to ST in domiciles despite of the absence of any previous community mobilization or training. Householders’ reports of domestic infestations typically have a high positive predictive value in endemic rural areas with high bug densities, and may be used for a rapid assessment of community-level infestation rates. Conversely, the negative predictive value of HN is usually poor at low bug densities, especially after effective interventions that nearly suppress infestations [19, 28, 30]. When intrusive triatomine species invade house premises without establishing bug colonies in them, the positive predictive value of HN may decline because house-dwellers may confound target and non-target vector species. A potentially relevant limitation of HN is related to the need to corroborate infestations before applying control measures [27], unless householders are encouraged to capture triatomines and hand them in at the local triatomine vigilance post [28, 30]. Another limitation of HN is linked to the eventual decline of their motivation to perform triatomine searches or notify the outcome during the extended vector surveillance phase when the density of the target vector becomes rare [28, 30]. The use of several detection methods can increase detectability of infestations [18, 48, 49], a key issue in elimination programmes. Moreover, the addition of ST or of any similar device may keep up householders’ motivation while providing strong evidence on the status of house infestation.

Although both TMC and ST captured nymphs and adults, the stage distribution of the *T. infestans* populations differed significantly between methods, as recorded with other devices [24]. The ST proportionally returned more early-instar nymphs, whereas TMC selectively returned later stages, as expected from its positive bias toward insects with a larger body mass [16]. In our study, chickens or lizards may have preyed on the triatomines, selectively on adults or latter nymph instars, that were caught by the ST.

The very low fraction of triatomines captured by ST that had colorless urine, combined with their lower W/L ratios than among those collected by TMC, suggests they were unfed bugs that were searching for a blood meal when they were trapped in bed legs, walls and chicken roosting places. The ST deployed in proximity to the resting places of hosts functioned as a baited trap that attracted triatomines, while providing a physical barrier that intercepted bugs while seeking for a blood meal. Therefore, STs may potentially reduce both low-density infestations and host-vector contact.

The blood-feeding intervals of the triatomines collected by TMC and ST were nearly 2–4 times greater than the time span over which the ST was exposed. However, domestic populations of *T. infestans* blood-fed every 3–4 days over the spring-summer period in other rural settings [43, 44]. Such high feeding rates are within the expected duration of adhesiveness of the ST. Periodic replacement of the sticky tape would allow longer follow-ups and the interception of host-seeking bugs under a less frequent schedule of blood-feeding.

The secondary vector *T. sordida* was only collected by TMC in one site, not by ST. This apparent absence is explained by the rare occurrence of *T. sordida* in domiciles, kitchens and storerooms [50] rather than by a species-bias of the trap. Other devices using sticky tapes readily caught several triatomine species [11, 25, 37, 40, 51].

Our study had some limitations. Lack of a gold standard for house or site infestation implied that our estimates of sensitivity are relative to the methods and definitions we used, and cannot be ascribed an absolute value. However, if the residual fraction of true infestations missed by the three methods were ascertained, it would equally increase all three denominators and not affect the ranking of methods. We did not evaluate the performance of ST in peridomestic ecotopes occupied by chickens, such as nests, trees and coops, which together with pig and goat corrals are frequently infested in the Chaco region. These ecotopes do not offer a suitable environment for the ST in its present form since moist and dust reduce its adhesiveness. Further studies are needed to apply this method in peridomestic habitats, and scaled-up trials are justified. Whether sticky tapes of different brands perform equally well remains to be investigated. Our mid-sized trial limited the power of the tests to detect significant differences between methods at α = 0.05. To achieve this goal in domiciles with 80% power, the required sample size is 400 houses.

The ST is a simple, socially acceptable vector detection method with several competitive advantages in reference to the more costly TMC for large-scale monitoring of domestic infestations: it is easy to transport, install, inspect, and can be readily transferred to vector control programmes, primary healthcare agents and householders alike. The STs are more apt for standardization than TMC searches, whose outcomes greatly influenced by the skill of vector control personnel and the habitats’ physical structure. The main limitations of the STs in its present format are the loss of adhesiveness by moist and dust accumulation. A preliminary assessment of the relative cost of ST to TMC revealed a highly favorable ratio for ST in the frame of a community-based control or elimination strategy, as it does not include the main direct costs of TMC (e.g. salary, transportation, *per diem* and dislodging sprays).

## Conclusions

The ST may effectively replace TMC for detecting *T. infestans* in domiciles, and is especially apt for early detection of low-density domestic infestations in the frame of community-based surveillance or elimination programmes. The emphasis on domestic triatomine surveillance is linked to where the vector-borne transmission of *T. cruzi* to humans mainly occurs. We envisage three key applications of the ST: (i) for decision making on whether a community or group of communities under sustained surveillance should be targeted again for full-coverage insecticide spraying-for the main domestic vectors such as *T. infestans*, vector control programmes indicated a new attack phase when house infestation rates exceeded 5% (control-action threshold); (ii) for vector elimination programmes whose ultimate goal is to fully suppress the occurrence of a target vector species in a well-defined area-detection of the very low-density infestations is of paramount relevance here; and (iii) to corroborate that domestic areas are free from domiciliated triatomines and the extant conditions are compatible with the interruption of vector-bone transmission. The combination of ST and HN may also promote community involvement and active responses toward the sustainable interruption of vector-borne transmission of Chagas disease.

## Supplementary information

**Additional file 1: Table S1.** Infestation with *Triatoma infestans* by sticky traps (ST), householdersʼ bug notifications (HN) and ST supplemented with HN (ST-HN) according to timed-manual collections (TMC).

**Additional file 2: Table S2.** Detection of infestation with *Triatoma infestans* by sticky traps (ST) and householdersʼ bug notifications (HN).

## Data Availability

Data supporting the conclusions of this article are included within the article and its additional files. The raw datasets generated during and/or analysed during the present study are available from the corresponding author upon reasonable request.

## Abbreviations

TMC: timed-manual collections
ST: sticky traps
HN: householders’ bug notification
