# Supplementary material for: Improved detection of house infestations with triatomines using sticky traps: a paired-comparison trial in the Argentine Chaco
Source: Parasit Vectors. 2020 Jan 14;13:26. doi: 10.1186/s13071-020-3891-z (PMC6961371; doi:10.1186/s13071-020-3891-z)
Supplement: Supplementary file 1 — Additional file 1: Table S1. Infestation with Triatoma infestans by sticky traps (ST), householdersʼ bug notifications (HN) and ST supplemented with HN (ST-HN) according to timed-manual collections (TMC). [file 13071_2020_3891_MOESM1_ESM.docx]

**Additional file 1: Table S1.** Infestation with *Triatoma infestans* by sticky traps (ST), householders' bug notifications (HN) and ST supplemented with HN (ST-HN) according to timed-manual collections (TMC).

|  |  |  | No. positive by | |  |  |  |
| --- | --- | --- | --- | --- | --- | --- | --- |
| Level | Alternative method | No. houses or sites | Both methods | Only TMC | Only alternative | No. negative by both methods | Exact McNemar’s test |
| House |  |  |  |  |  |  |  |
|  | ST | 54 | 13 | 6 | 8 | 27 | *P* = 0.791 |
|  | HN^a^ | 49 | 9 | 9 | 7 | 24 | *P* = 0.804 |
|  | ST-HN | 54 | 15 | 4 | 12 | 23 | *P* = 0.077 |
| Domiciles |  |  |  |  |  |  |  |
|  | ST | 51 | 5 | 3 | 6 | 37 | *P* = 0.508 |
|  | HN | 47 | 6 | 1 | 9 | 31 | *P* = 0.022 |
|  | ST-HN | 51 | 6 | 2 | 11 | 32 | *P* = 0.023 |
| Kitchens and store-rooms | | |  |  |  |  |  |
|  | ST | 52 | 8 | 6 | 3 | 35 | *P* = 0.508 |
|  | HN | 47 | 3 | 9 | 0 | 31 | *P* = 0.004 |
|  | ST-HN | 52 | 8 | 6 | 3 | 35 | *P* = 0.508 |

a. Householders' bug notification was not registered in five houses.
