# Supplementary material for: Improved detection of house infestations with triatomines using sticky traps: a paired-comparison trial in the Argentine Chaco
Source: Parasit Vectors. 2020 Jan 14;13:26. doi: 10.1186/s13071-020-3891-z (PMC6961371; doi:10.1186/s13071-020-3891-z)
Supplement: Supplementary file 2 — Additional file 2: Table S2. Detection of infestation with Triatoma infestans by sticky traps (ST) and householdersʼ bug notifications (HN). [file 13071_2020_3891_MOESM2_ESM.docx]

**Additional file 2: Table S2.** Detection of infestation with *Triatoma infestans* by sticky traps (ST) and householders' bug notifications (HN).

|  |  | |  | No. positive by | | | | |  | | |  | |  | |
| --- | --- | --- | --- | --- | --- | --- | --- | --- | --- | --- | --- | --- | --- | --- | --- |
| Ecotope |  | No. of sites | | Both methods | | Only HN | | Only ST | | | No. negative by both methods | | Exact McNemar’s test | |  |
| Domiciles^a^ | | 47 | | | 9 | 6 | 2 | | | 30 | | | *P* = 0.289 | | |
| Kitchens and storerooms^b^ | | 47 | | | 3 | 0 | 6 | | | 38 | | | *P* = 0.031 | | |
| All sites | | 94 | | | 12 | 6 | 8 | | | 68 | | | *P* = 0.791 | | |

a. The data were not available for four sites from 5 houses.

b. The data were not available for five sites from 5 houses.
